# Propylene Phenoxetol (1-Phenoxypropan-2-ol): Its Use as an Anesthetic for Echinoderms

**DOI:** 10.3390/biology15212007

**Published:** 2026-11-05

**Authors:** Iain C. Wilkie

**Affiliations:** School of Biodiversity, One Health and Veterinary Medicine, University of Glasgow, Glasgow G12 8QQ, UK; iain.wilkie@glasgow.ac.uk

**Keywords:** aquaculture, invertebrate welfare, magnesium chloride, mutable collagenous tissue, phenoxyisopropanol, 1-phenoxy-2-propanol, propylene glycol phenyl ether, sea cucumber autolysis

## Abstract

Propylene phenoxetol is the trade name of the chemical compound that has the systematic name 1-phenoxypropan-2-ol. Used as an anesthetic agent for aquatic animals—mainly invertebrates—for over 70 years, it has been employed most extensively for all five classes of the phylum Echinodermata both to narcotize whole animals—reversibly or terminally—and as a pharmacological agent for blocking neural conduction in isolated tissue preparations. Despite this long history, there is evidence that since the 1990s, there has been a sustained decline in propylene phenoxetol usage, which may be partly the result of an accumulation of misinformation about its chemical identity, physical properties, and physiological actions. This review surveys the use of propylene phenoxetol as an anesthetic for echinoderms and other animals, outlines the history of misinformation concerning its nature and qualities, and corrects that misinformation. Deficiencies in current knowledge are outlined, with the ultimate aim of encouraging further research to explore its potential as an option for the anesthetization of echinoderm subjects.

## Simple Summary

Propylene phenoxetol is the trade name of the compound whose standardized chemical name is 1-phenoxypropan-2-ol. For over 70 years it has been used to anesthetize aquatic animals, particularly members of the phylum Echinodermata (starfish, sea urchins and their close relatives). It appears that since the 1990s, there has been a notable drop in its usage, which may have been caused partly by incorrect information about its identity, properties (solubility and volatility), and effects on animals. This review surveys the employment of propylene phenoxetol as an anesthetic for echinoderms, details the misinformation that has circulated about it, corrects that misinformation, and exposes gaps in current knowledge.

## 1. Introduction

The welfare of invertebrates is an emerging topic. This is due in part to their increasing exploitation as a human food resource [1,2,3] and as model organisms for experimental biology and biomedical research [4,5,6,7]. Perhaps more importantly, it is also due to accumulating evidence that invertebrates have greater sentience and cognitive ability than suspected [8,9] and that some of them have much more sophisticated nervous systems than previously inferred. For example, many species belonging to the phylum Echinodermata show abundant evidence for memory and both associative and non-associative learning [10,11], which is discordant with the persisting non-specialist view that they have simple nervous systems (see, e.g., refs. [12,13,14]). On the contrary, the recent discovery that the genetic organization of the juvenile sea urchin nervous system resembles that of the vertebrate brain suggests that echinoderm nervous systems represent an “all-brain” state rather than the previously assumed “no-brain” state [15].

In light of these considerations, it is obvious that anesthetic procedures should be playing an increasingly important role in the humane management of invertebrates in scientific and commercial contexts. A wide range of anesthetic agents has been applied to invertebrates [9]. With regard to aquatic invertebrates, one of the most widely used is the subject of this review—propylene phenoxetol. Propylene phenoxetol is the trade name of the chemical compound that has the systematic name 1-phenoxypropan-2-ol [16]. In view of reservations about its identity, which are discussed in Section 3.1.1, it should be noted that, unless stated otherwise, in all instances where the name propylene phenoxetol (and its abbreviation—PP) is cited below without quotation marks, the present author could verify that the source publication refers to 1-phenoxypropan-2-ol, usually because the correct systematic name, or a correct synonym of the systematic name, is explicitly acknowledged or because the commercial supplier is stated.

PP appears to have been first synthesized as an analgesic and pharmaceutical intermediate around 1915 [17] and was then used as an antibacterial agent against Gram-negative organisms [18,19] and as an antifungal agent [20]. It has also been employed as a post-fixation preservative for animal material [21,22,23]; currently, it is widely incorporated into cosmetics and soaps as a sterilizing and preserving agent, and into paints, inks, coatings, and other products as a solvent [16,24,25]. It has been utilized as an anesthetic for a range of aquatic animals—mainly marine invertebrates—for over 70 years and has been applied most extensively to all five classes of the phylum Echinodermata, both to narcotize whole animals—reversibly or terminally—and as a pharmacological agent for blocking neural conduction in isolated tissue and organ preparations. Despite this long history, there has been a sustained decline in propylene phenoxetol usage as an echinoderm anesthetic, which may be partly the result of an accumulation of misinformation about its chemical identity, physical properties, and physiological actions.

This review surveys the use of PP as an anesthetic, first, to provide context, for animals other than echinoderms then for echinoderms. It outlines the history of misinformation concerning the nature of PP and its qualities and corrects that misinformation. Deficiencies in current knowledge of the anesthetic action of PP on echinoderms are also outlined, with the ultimate aim of encouraging further research to explore its potential as an option for the anesthetization of echinoderm subjects for experimental and commercial purposes. In the following text, “anesthetic” and related words refer to any chemical compound that, applied to intact animals or surgically excised in vitro preparations, renders them immobile and unresponsive to external stimulation or any neurostimulatory treatment. No distinction is made between “anesthetic”, “narcotic” and “relaxing agent”. All PP concentrations are given as percentages and have been converted as necessary (assuming 0.1% ≈ 6.6 mmol L^−1^), i.e., when the original source provides mass or molar concentrations.

## 2. Use of PP as an Anesthetic Agent

### 2.1. Animals Other than Echinoderms

#### 2.1.1. Mollusca

The earliest report of PP being used as an anesthetic agent appears to be that of Owen [19], who immersed several species of bivalve molluscs in a seawater solution of PP in order to “relax” them prior to fixation for histological purposes. The final concentration of PP employed was not stated. It was noted that specimens of the cockle, *Cardium edule*, were completely relaxed after immersion for 30 min and that, if returned to fresh seawater, relaxed animals recovered fully. PP was subsequently advocated in a commercial context for the reversible anesthesia of pearl oysters to reduce stress on them during the surgical insertion of the “bead” (or “nucleus”) and mantle graft or to permit examination and biopsy of the gonads [26,27,28,29,30]. These authors used PP concentrations of 0.15–0.35%; relaxation required immersion for up to 40 min, and full recovery with no ill effects took 30–120 min. In an experimental context, the usefulness of PP at concentrations of 0.24% and above for inducing reversible anesthesia before surgery for neurobiological investigations was demonstrated with the sea slugs *Tritonia diomedea* and *Hermissenda crassicornis* (Gastropoda, Nudibranchia) [31,32]. PP has been used to narcotize chitons and bivalves in macrobenthos samples before fixation [33,34]. PP (wrongly referred to as “2-phenoxyethanol”) is mentioned briefly in a survey of around 20 anesthetic agents for molluscs [35].

#### 2.1.2. Nematoda

PP has been recommended for reversible anesthetization of nematodes to permit the microscopical examination of living animals for identification and other purposes [36,37]. Townshend [37] found that the optimal concentration for three species was 0.25–0.5% in tap water, with the time taken to achieve anesthesia varying from ca. 0.5 to 4 h, and recovery taking ca. 0.5–9 h. Nelson & Riddle [38] used 0.05–0.4% PP to anesthetize *Caenorhabditis elegans* larvae prior to the laser ablation of specific cells in their secretory–excretory system; the larvae were allowed to recover for follow-up observation.

#### 2.1.3. Chordata, Teleostei

Anesthetics are widely used to facilitate scientific research on teleost fish that involves interventions such as surgery in preparation for physiological investigations and to minimize stress on fish during aquaculture-related procedures, such as weighing, vaccination, and transport [39,40]. Bagenal [40] found that plaice (*Pleuronectes platessa*) were completely anesthetized by immersion for 30 min in 0.05% “propylene phenoxetol” (source unstated), with recovery taking as little as 6 min. In an investigation of the effects of a number of anesthetics on Mozambique tilapia (*Oreochromis mossambicus*), 0.08% PP produced full anesthesia in 19 min and, after return to fresh water, opercular activity restarted in 1 min and full recovery took 15 min (all values are means); the equivalent values for 0.08% MS-222 (tricaine methanesulfonate) were 1 min, 10 min and 17 min, respectively, and for 0.08% chlorbutol (chloretone) they were 12 min, 5 min, and 55 min [41]. Czerniak et al. [42] suggested that PP might be preferable as a fish anesthetic to the more commonly used 2-phenoxyethanol, since, although the time to achieve deep anesthesia in common carp (*Cyprinus carpio*) at the minimum effective dose was slightly shorter for the latter, the recovery time after PP was longer, thus reducing the risk of “early awakening” associated with 2-phenoxyethanol. However, hematological data indicated that PP-induced anesthesia causes a moderate, temporary stress response in these fish, which is more severe than that reported for 2-phenoxyethanol [43].

PP is not amongst the roughly 40 anesthetic agents for fish surveyed by Ross & Ross [35] and is neither discussed by Ackerman et al. [39] nor included in their list of 12 recommended fish anesthetics.

#### 2.1.4. Other Animals

PP has been used to anesthetize bryozoans. Lewin et al. [44] submerged colonies of *Flustrellidra hispida* in seawater containing 0.1% PP to permit the detachment of lophophores for transcriptomic analysis. Even after anesthetization for 3–4 h, colonies returned to fresh seawater recovered “quickly”, with their remaining lophophores resuming normal feeding and defensive activities. PP (0.03%) has been used on ascidians as a pre-fixation relaxant [45], and possibly on annelids: “propylene phenoxetol” (source unstated) at concentrations of 0.015–0.15% has been cited as a pre-fixation and pre-dissection anesthetic for clitellates [46,47,48].

Like molluscs, fish, and echinoderms, crustaceans are a major group of animals whose welfare issues relate to both their use as experimental models in biological research and their commercial exploitation in the aquaculture and fishing sectors [49]. PP was not mentioned in the survey by Ross & Ross [35] of over 20 crustacean anesthetics, or in a recent comprehensive review of decapod crustacean anesthesia, also covering around 20 different agents [50]. The present author has found no verifiable example of PP being used as a crustacean anesthetic (see also refs. [9,51]).

No literature has been found signifying its use, verified or unverified, as an anesthetic for members of any other non-echinoderm phyla (see also ref. [9]).

#### 2.1.5. Summary

PP has been exploited occasionally as an anesthetic for a limited assortment of aquatic, mainly invertebrate, non-echinoderm animals, in the context of both scientific research and commercial aquaculture. It has been recommended for use when handling pearl oysters and certain teleost fish but is not the favored anesthetic agent for any other non-echinoderm animal groups. These comments would apply even if all the unverified citations of “propylene phenoxetol” were confirmed as referring to PP.

### 2.2. Echinoderms

#### 2.2.1. Use of PP as an Echinoderm Anesthetic

**Figure 1:**
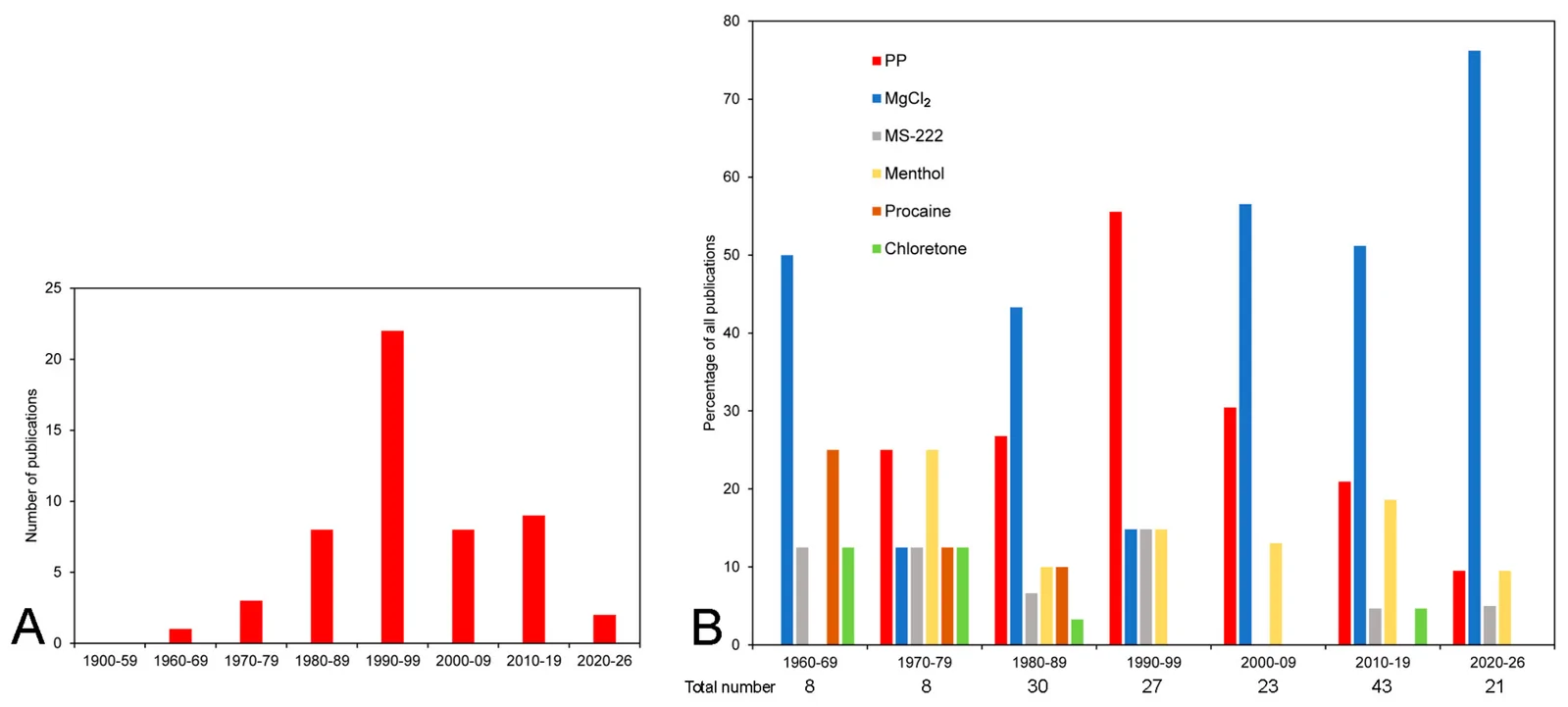
Echinoderm anesthetic agents: analysis of the literature. (**A**) Number of publications (1900–2026) reporting investigations that refer to the use of propylene phenoxetol to successfully induce anesthesia in echinoderm subjects (completed 14 September 2026). Data from Table S1. The majority (77%) of these publications were identified in an initial manual search of the author’s own library of echinoderm literature assembled over more than 50 years. This was supplemented by searches conducted via the Scopus database (https://www.scopus.com), Google, and Google Scholar, using “propylene phenoxetol” or its synonyms alone or with word combinations of “propylene phenoxetol” and “Echinodermata” or “Asteroidea” or “Crinoidea”, etc., and “relaxant”. Theses were excluded. (**B**) Relative citation rates of the six most commonly used echinoderm anesthetics. The relative citation rate is the number of publications referring to a particular agent expressed as a percentage of the total number of identified publications (below the chart) referring to all six agents in a given time period. Publications were identified as described above, but excluding the Google Scholar search.

The history of PP as an echinoderm anesthetic may have started around 1960 when a paper by Nichols [52] mentioned the narcotization of specimens of the feather star *Antedon bifida* in a seawater solution (concentration not given) of “propylene phenoxytol” [sic] before they were fixed for histological processing. The earliest verified echinoderm application of PP involved the anesthetization of lantern muscles in the sea urchin *Echinus esculentus* (concentration not given) prior to fixation for ultrastructural investigation reported in 1966 [53]. Figure 1A, which is derived from aliterature search (Table S1; refs. [52,53,54,55,56,57,58,59,60,61,62,63,64,65,66,67,68,69,70,71,72,73,74,75,76,77,78,79,80,81,82,83,84,85,86,87,88,89,90,91,92,93,94,95,96,97,98,99,100,101,102,103,104,105,106,107,108,109,110,111,112]), shows that the incidence of publications citing PP use on echinoderms rose after the 1960s, reached a maximum in the 1990s, and then dropped. The relative citation rate follows a similar pattern when PP is compared with five other commonly used echinoderm anesthetics: it peaked in the 1990s and declined steeply thereafter (Figure 1B), even though the total number of publications referring to these compounds did not show such a trend. It is a reasonable proposition that publication rate may reflect, at least roughly, research activity. Figure 1B may therefore indicate that relative PP usage fell after the 1990s. Fading interest in PP is also suggested by its omission from recent publications referring to echinoderm anesthesia [113,114]. None of the other five compounds replicate the pattern shown by PP. It is notable that the citation rate for magnesium chloride has not declined since the 1990s (Figure 1B).

**Figure 2:**
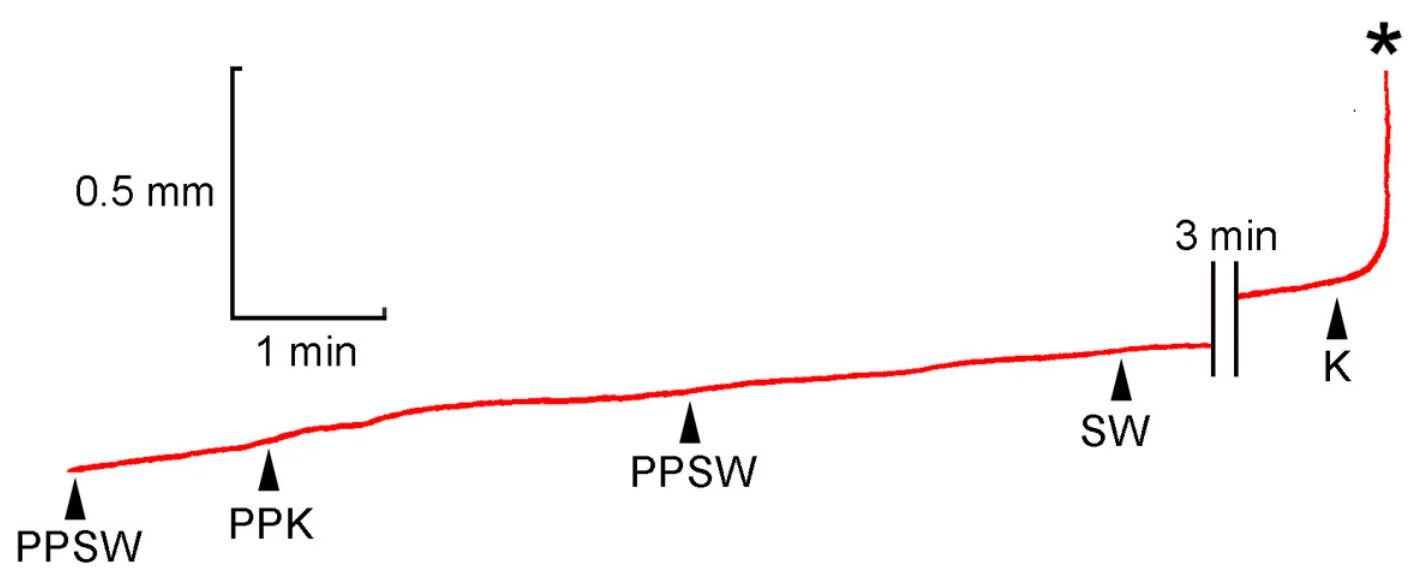
Example of the anesthetic action of PP on an isolated preparation. Elevated K^+^ ion concentrations normally cause a decrease in the viscosity of the aboral disk integument (ADI) of the brittle star *Ophiocomella ophiactoides*, which can be blocked by PP. The red curve represents a recording of extension against time of an ADI sample elongating under a constant tensile load. At the start of the recording, the preparation is immersed in 0.1% PP in seawater (PPSW). Replacement of this with isotonic (560 mmol L^−1^) potassium chloride solution containing 0.1% PP (PPK) has no effect. After immersion in seawater alone (SW) for ca. 4 min, treatment with 560 mmol L^−1^ potassium chloride solution alone (K) results in a drastic decrease in the viscosity of the sample, which is manifested as a sudden increase in the extension rate culminating in rupture (asterisk). Data from ref. [107].

During the past 50 years, PP has been employed to anesthetize both whole animals and isolated in vitro preparations from all five echinoderm classes (Table S1). Table 1 provides specific examples from each class. Whole animals have been anesthetized with subsequent recovery in the course of behavioral experiments (e.g., ref. [105]). However, most whole animals have been anesthetized irreversibly, the commonest purpose being to permit the excision of isolated preparations for physiological studies; 33 articles citing PP refer to such studies, of which 22 (i.e., 67%) were intended for the investigation of components consisting of mutable collagenous tissue (MCT: see ref. [115] for a recent review). The action of PP on isolated preparations has been demonstrated by its inhibition of responses to neuroactive agents such as elevated K^+^ concentrations (Figure 2).

No determination of the dose–response relationship or optimal induction and recovery times for any echinoderm subject—either intact individuals or isolated preparations—has been reported. Neither has there been any investigation of the efficacy of PP use on whole animals for achieving different endpoints, such as immobilization (capacity for locomotion suppressed; responsiveness to external stimuli reduced), muscular relaxation (muscle contractility suppressed) and reversible deep anesthesia (capacity for locomotion suppressed; muscle contractility suppressed; responsiveness to external stimuli eliminated). Criteria to determine anesthesia and recovery have not been described. In 42 (82%) of 51 articles referring to verified PP use to induce anesthesia and stating the concentration employed, PP was applied at a concentration of 0.1% (6.6 mmol L^−1^) in natural seawater or artificial seawater; the other stated concentrations ranged from 0.15% to 2% and “saturated”. Goundar et al. [117] tested only PP concentrations of 0.02% and below, which had no measurable narcotic effect on the sea cucumber *Holothuria scabra*. It is highly likely that the optimal induction time (the minimal duration of exposure to anesthetic required to induce unresponsiveness) varies significantly between intact specimens of different species and between different isolated preparations. Whilst reported immersion times vary between 10 min and 300 min (most being within the 30–60 min range), it was observed that intact individuals of the feather star *Antedon mediterranea* were rendered unresponsive “in seconds” [68]. Less information is available on recovery times; most intact animals and isolated preparations were “washed” in anesthetic-free seawater for 15–60 min, though experimental evidence suggests that physiological recovery can occur in much shorter timescales (Figure 2).

An analysis of the literature indicated that magnesium chloride (MgCl_2_) is by far the most frequently cited echinoderm anesthetic (Figure 1B). MgCl_2_ has been used in several formulations, the most physiologically rational of which is a 370 mmol L^−1^ aqueous solution (which is isosmotic with seawater [118]) either by itself or mixed with seawater (e.g., refs. [119,120]). More concentrated aqueous solutions (e.g., 1 mol L^−1^) and solutions of MgCl_2_ in seawater (resulting, e.g., in a final concentration of 740 mmol L^−1^) have also been employed [121,122]. Since such media are hyperosmotic with respect to seawater and echinoderm celomic fluid [118], they will inevitably cause osmotic damage to tissues, which matters if the anesthetized animals are intended for physiological or morphological (histological and ultrastructural) research. MgCl_2_ is also problematic as an echinoderm anesthetic because of evidence that, in addition to influencing neural conduction [123], Mg^2+^ ions contribute directly to the structural integrity and mechanical behavior of the extracellular matrix of echinoderm MCT [124,125], a tissue that is ubiquitous in all five echinoderm classes [115].

The reason for the marked reduction in the relative citation rate, and therefore possibly usage, of PP since the 1990s is unclear. If there was a decline in its usage, it is possible that this was partly a consequence of shifts in the focus of echinoderm research over the past 30 years. However, no correlational trends emerged from a comparison of the different investigation categories included in Table S1, although, being obviously limited to research involving anesthetic agents, this is not conclusive. Another factor that may have contributed to the putative decline in relative PP usage is the misinformation about PP that has been accumulating for almost as long as it has been employed as an echinoderm anesthetic and that may have discouraged its use.

#### 2.2.2. Summary

From the 1960s onwards, PP has been used extensively as an anesthetic for all five classes of echinoderms, both to narcotize whole animals and as a pharmacological agent for blocking neural conduction in isolated tissue preparations. Information is available on the induction and recovery times for PP with regard to many echinoderm subjects, although no determination of the dose–response relationship or optimal induction and recovery times has been reported. A comparison with other commonly used agents suggests that the relative citation rate of PP as an echinoderm anesthetic peaked in the 1990s and subsequently underwent a steep and sustained decline.

## 3. Misinformation About PP

### 3.1. Chemical Identity and Physical Properties of PP

#### 3.1.1. Chemical Identity

Two facets of the identity of “propylene phenoxetol” need to be addressed: (1) the identity of the compound given this name by its manufacturers and suppliers and (2) the identity of the compound given this name in scientific publications.

**Figure 3:**
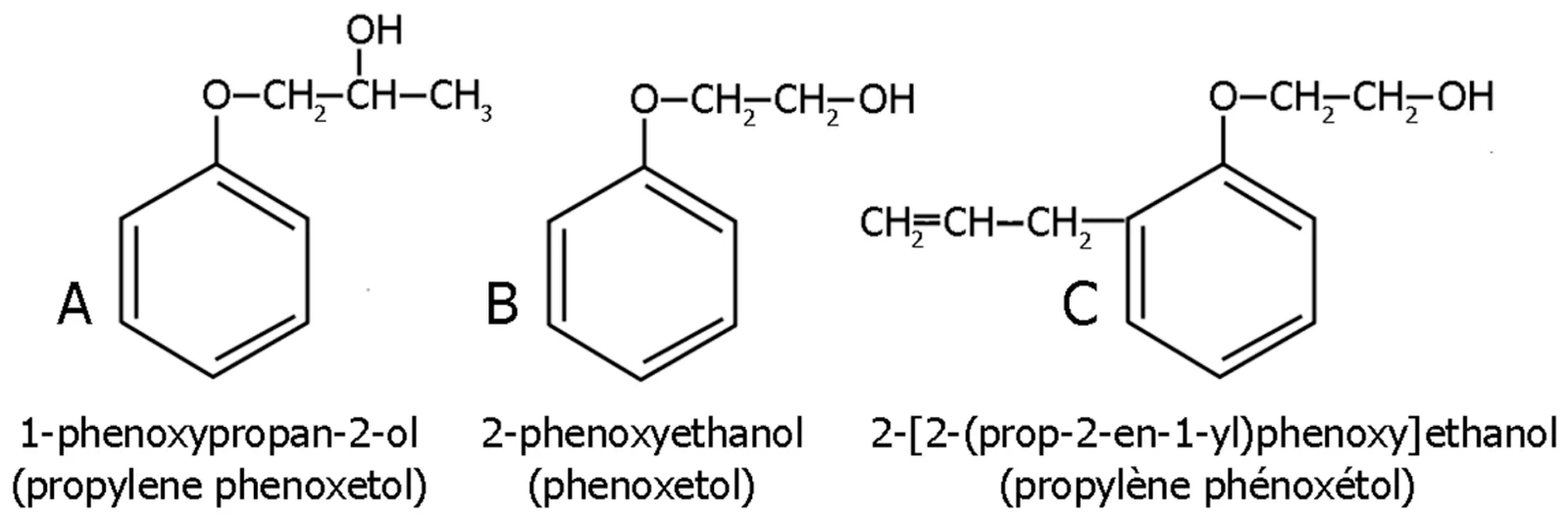
Structural formulae, systematic (IUPAC) names (not in parentheses) and non-systematic names (in parentheses). (**A**,**B**) Information from ref. [16]. (**C**) Information from refs. [116,130].

“Propylene phenoxetol” is an unregistered trade name for the chemical compound that has the systematic name 1-phenoxypropan-2-ol (as recommended by IUPAC: International Union of Pure and Applied Chemistry [126]) (Figure 3A). It has the CAS (Chemical Abstracts Service) number 770-35-4 and its molecular weight is 152.19 [16]. Its several non-systematic synonyms include 1-phenoxy-2-propanol, phenoxyisopropanol and propylene glycol phenyl ether [16]. A German patent for its synthesis, under the name “Propylenglykol-(1)-phenyläther(2)”, was published in 1915; it and its isomer were intended for use as analgesic agents and as intermediates for pharmaceutical preparations [17]. The trade name was first used by the chemical manufacturer Nipa Laboratories Ltd., UK, not later than 1949 [18]. However, the “phenoxetol” component of the name was invented by H. Berry in 1944 [127] to denote a compound that has the IUPAC name 2-phenoxyethanol [16] (Figure 3B). “Phenoxetol” is a registered trademark with a filing date of the 26th of November 1946 and registration date of the 9th of November 1948 [128]. As the registrant was Nipa Laboratories Ltd., Cardiff, UK [128], this company is likely to have introduced the variant “propylene phenoxetol” for another of its products. A published product list for Nipa Laboratories Ltd. confirms that this name applies to “1-phenoxy-2-propanol” (one of the synonyms of 1-phenoxypropan-2-ol) [129]. Nipa Laboratories Ltd. became part of the Clariant company in 2000 and was dissolved in 2022. Current suppliers of propylene phenoxetol (e.g., Merck, Darmstadt, Germany; Thermo Fisher Scientific, Waltham, MA, USA; VWR Chemicals BDH, Radnor, PA, USA) include the systematic name, CAS number, and molecular weight in their product information, leaving no doubt as to its chemical identity.

Regarding the use of “propylene phenoxetol” in scientific publications, an early problem resulted from the application by Laborit et al. [130,131,132] of the French version of the name—propylène phénoxétol—to a compound that was not 1-phenoxypropan-2-ol. Based on the structural formula provided by these authors (Figure 3C) [130], their compound would have the systematic name 2-[2-(prop-2-en-1-yl)phenoxy]ethanol [116]. The assumption by Hill [85] that “propylene phenoxetol” and the “propylène phénoxétol” of Laborit et al. referred to the same compound led to confusion about the biological actions of the former, which is explained in Section 3.2. It was also one reason why O’Neill [116] first expressed doubt about the identity of “propylene phenoxetol” in the earlier literature. O’Neill asserted that it was “impossible to ascertain” which chemical had been employed by previous authors. This was an overgeneralization: O’Neill herself admitted that (1) some authors had cited their source as Nipa Laboratories Ltd., (2) the Martindale Pharmacopeia of 1977 [133] listed Nipa Laboratories Ltd. as the manufacturer of “propylene phenoxetol”, and (3) the Chemical Abstracts Index Guide of the time listed “1-phenoxy propane-2-ol” [sic] as its IUPAC name. Furthermore, even where authors did not state the source or correct systematic name of the agent, it is unreasonable to question their veracity unless there is other cause for doubt. A recurring example of the latter is the presumption that “propylene phenoxetol” is synonymous with “phenoxetol” or “2-phenoxyethanol” (see, e.g., refs. [35,134,135,136], which have not been included in Table S1). An additional factor contributing to O’Neill’s suspicions was the status of “propylene phenoxetol” as a “trivial” non-systematic name, the insinuation being that this was an inherent source of ambiguity, a view endorsed by Sendall [137]. However, numerous commonly used non-systematic names of organic compounds are specific and unambiguous (e.g., acetone, formaldehyde, paracetamol, and nitroglycerin); indeed, some are acceptable under IUPAC rules (e.g., acetic acid, benzene, styrene, and urea) [126]. In general, the supposed uncertainty about the identity of “propylene phenoxetol” in the anglophone literature seems ill-founded, though it continues to be articulated [138].

#### 3.1.2. Physical Properties

It has been stated repeatedly and erroneously that PP is immiscible in water (see, e.g., refs. [116,138,139,140]). PP does, however, dissolve in water, albeit slowly [21], and it has a solubility of 15.1 g L^−1^ at 20 °C [16].

O’Neill [116] claimed that PP is “a volatile liquid” and that its volatility, together with its supposed immiscibility in water, made it difficult to maintain known concentrations of the agent—comments that have been echoed in the recent literature [138]. PP has a vapor pressure of 0.001 kPa at 20 °C and a relative evaporation rate of <0.01 (*n*-butyl acetate = 1.0) [141]. Water has a vapor pressure of 2.34 kPa and a relative evaporation rate of 0.3 [142,143]. PP is therefore considerably less volatile than water and, like water, is classified as “slow evaporating” [143,144].

#### 3.1.3. Summary

“Propylene phenoxetol” has been in use since the late 1940s as an unregistered trade name for 1-phenoxypropan-2-ol. In the 1990s, doubt was cast on the identity of the compound(s) assigned this name in biological publications, mainly on the insubstantial grounds that it was a non-systematic name and that there had been a single case of it being applied to a substance other than 1-phenoxypropan-2-ol in the French scientific literature. It has been wrongly stated that PP is immiscible in water, that it is a volatile substance, and that, due to these attributes, it is difficult to maintain known concentrations of it. PP, however, has a solubility in water of 15.1 g L^−1^ and is classified as “slow evaporating”, since its vapor pressure and relative evaporation rate are lower than the equivalents for water.

### 3.2. Physiological Actions of PP

#### 3.2.1. Antioxidant Action

**Figure 4:**
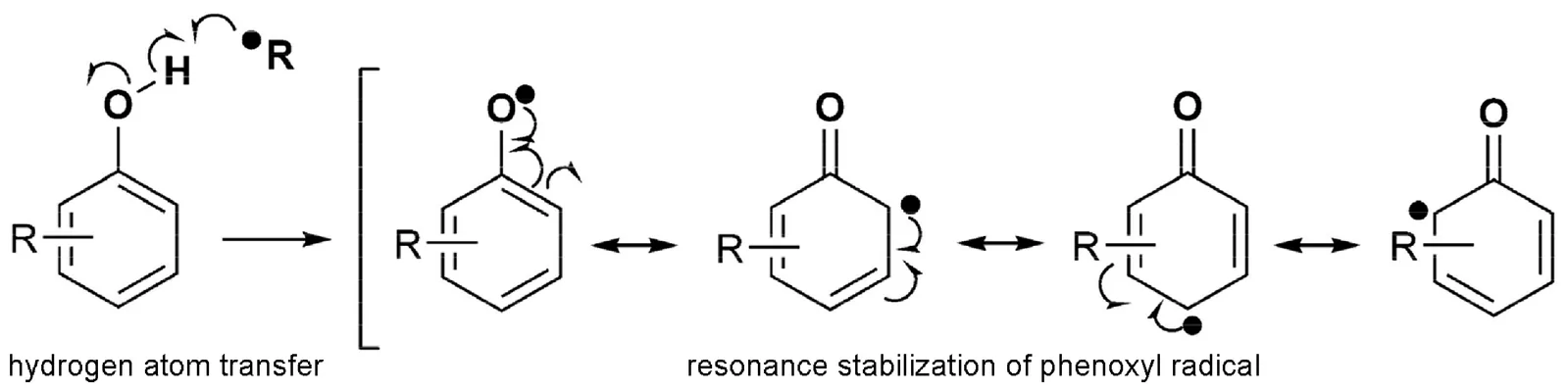
Antioxidant mechanism of phenolic compounds. A free radical (^•^R) is neutralized by the transfer of a hydrogen atom from a hydroxyl group directly connected to the benzene ring. The phenol becomes a phenoxyl radical. This, however, is relatively unreactive because it is stabilized by the resonance delocalization of the unpaired electron (•) around the benzene ring. Adapted from ref. [147].

It has been claimed that PP is an antioxidant. This was first asserted in 1966 by R.B. Hill [85], who had observed that, as well as “relaxing” sea cucumbers, “propylene phenoxetol” blocked the mechanically provoked autolysis (“liquefaction”) of sea cucumber body wall. Hill speculated that the latter effect, which was confirmed in a subsequent experimental investigation [88], might be due to the antioxidant action of the compound, citing Laborit et al. [130] as the authority corroborating its antioxidant activity. Whilst Laborit et al. [130] did refer to “propylène phénoxétol” and did consider this to be an antioxidant, their compound is not 1-phenoxypropan-2-ol, as discussed above (Section 3.1.1). Hill repeated the assertion that PP is an antioxidant in later papers [88,99] (the latter also providing confirmation that Hill’s compound was 1-phenoxypropan-2-ol). O’Neill [116] commented that it “was originally used as an antioxidant and preservative for histological specimens”, citing a 1949 paper by Gough and Wentworth [18]. This comment, however, was based on the presumption that an effect noted by these authors—the restoration of the natural color to blood pigments in formalin-fixed lung tissue—resulted from antioxidant activity; Gough and Wentworth did not themselves discuss the chemical reactions that might be responsible for the effect and did not use the term “antioxidant”. The ability of a 1% aqueous PP solution to preserve some of the natural color of blood in formalin-fixed animals and organs was confirmed by Owen & Steedman [21], who also did not discuss the chemistry of the effect. There is in fact no empirical evidence that PP is an antioxidant. Furthermore, its structure suggests that, if it does have any antioxidant activity, this is likely to be fairly weak [145]. Not being a phenolic compound, PP lacks hydroxyl groups directly attached to the aromatic ring; such hydroxyls, by transferring hydrogen atoms to, and thereby neutralizing, free radicals, are responsible for the potent antioxidant activity of phenols [146,147] (Figure 4).

#### 3.2.2. Anesthetic Action

As recounted in Section 1 and Section 2, PP was first synthesized for therapeutic use as an analgesic agent, and its anesthetic action on animals has been recognized and exploited since the 1950s. According to McKay and Hartzband [139], PP-induced anesthesia results from the inhibition of acetylcholine synthesis. As the source of this information, McKay and Hartzband cited Laborit et al., who believed that, through their antioxidant activity, “propylène phénoxétol” and other compounds produced certain physiological effects in mammals by inhibiting acetylcholine synthesis [131,132]. As stated above (Section 3.1.1 and Section 3.2.1), the “propylène phénoxétol” of Laborit et al. is not 1-phenoxypropan-2-ol. Rotllant et al. [49] more recently repeated the statement that PP is an acetylcholine synthesis inhibitor, citing Wyeth et al. [32]. Since Wyeth et al. [32] did not make such a claim, the likely source was McKay and Hartzband [139], which is included in Rotllant et al.’s reference list. There is no evidence that PP inhibits acetylcholine synthesis.

**Figure 5:**
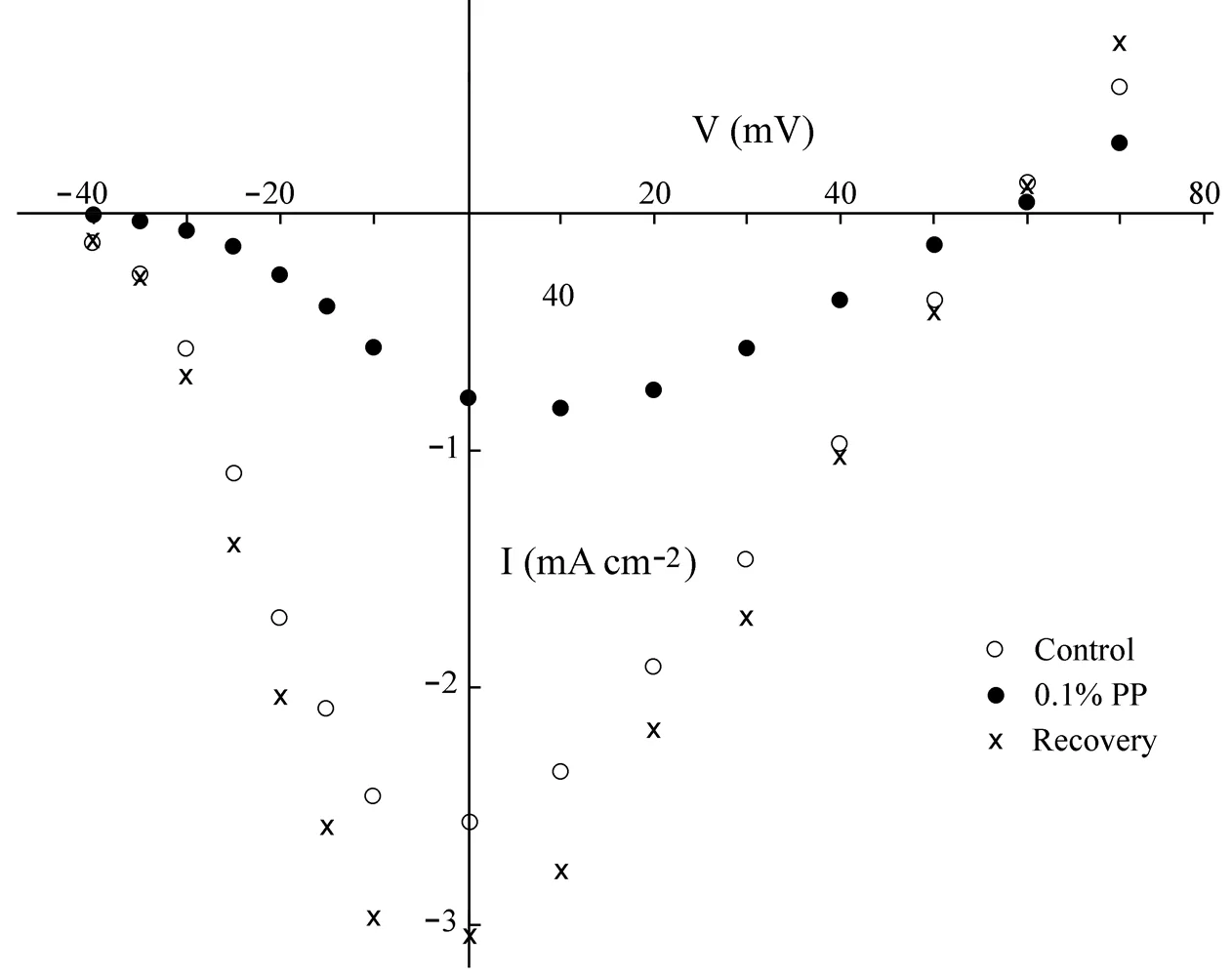
Electrophysiological action of PP. Effect of 0.1% (6.6 mmol L^−1^) PP on sodium currents in a voltage-clamped squid (*Loligo forbesii*) giant axon. *V* is the membrane potential during a test pulse, and *I* is the maximum sodium current measured during each pulse. Inward current is represented by negative values of *I*. Data courtesy of D.A. Haydon (pers. comm.).

PP has been shown to block neural conduction in several invertebrate preparations. By means of voltage clamp experiments, it has been demonstrated that 0.1% (6.6 mmol L^−1^) PP suppresses the sodium current in the squid (*Loligo forbesii*) giant axon sufficiently to block impulse propagation (Figure 5) [148]. The concentration of PP used was quite close to the nerve block concentration for the squid axon of the local anesthetic benzocaine (ca. 1 mmol L^−1^ [149]). Extracellular recording from the exposed radial nerve cord in the arm of the brittle star *Ophiura ophiura* was used to determine the electrophysiological effect of 0.1% PP (for the methodology, see ref. [150]). It was found that there was a burst of neural activity when PP was first introduced, with activity in giant axons and smaller axons ceasing over a period of 5 min; neural activity was largely restored after a 30 min washout [151]. In a comprehensive neurophysiological investigation of the nudibranch mollusc *Hermissenda crassicornis*, which included intracellular recording from a range of nerves, Wyeth et al. [32] showed that 0.03–0.5% (2–32 mmol L^−1^) PP had a dose-responsive effect on action potential duration, amplitude, and counts. PP caused an initial transient increase in resting potential accompanied by an increased frequency of postsynaptic potentials and action potentials (as observed by Cobb in the brittle star radial nerve), with the action potentials then ceasing entirely over several minutes. Activity was restored completely after removal of the anesthetic. Available evidence thus indicates that PP is a local anesthetic agent that inhibits sodium currents across axonal membranes [152].

#### 3.2.3. Summary

The attribution of antioxidant activity to PP originated from an overinterpretation of observations on its preservative properties and from the assumption that it was the same compound as that called “propylène phénoxétol” by certain French investigators, which was considered by them to be an antioxidant but which is not 1-phenoxypropan-2-ol. The same assumption was responsible for the misinformation that PP acts as an anesthetic by blocking acetylcholine synthesis. Neurophysiological investigations of its effect on squid, brittle star, and nudibranch preparations indicate that PP is a local anesthetic that inhibits sodium currents across axonal membranes.

## 4. Conclusions

### 4.1. Overview

“Propylene phenoxetol” has been the trade name for 1-phenoxypropan-2-ol since the 1940s. It appears to have been first synthesized in 1915 as an analgesic and pharmaceutical intermediate, then used as an antibacterial and antifungal agent, and is now employed widely as an industrial solvent and a preservative in cosmetics and soaps. It is soluble in water (up to a concentration of 15.1 g L^−1^), has low volatility, and has been shown to act as a local anesthetic that interrupts nervous conduction by blocking sodium channels in axonal membranes. It has been used as an anesthetic for aquatic animals since the 1950s. An analysis of the literature suggests that, although there may have been no decline in research activity involving echinoderm anesthesia since the 1980s, reliance on PP for this purpose may have peaked in the 1990s and fallen thereafter. If there was a post-1990s decline in its usage, it is possible that misinformation about its chemical identity, physical properties, and physiological actions was a contributory factor.

### 4.2. Future Applications of PP

The literature search suggested that PP has been used as an echinoderm anesthetic only in the context of non-applied research. No publications were found that cited its employment either for procedures requiring the narcotization of fished or farmed sea cucumbers or sea urchins or for aquacultural or fisheries research on these animals (Table S1). It is hoped that, by establishing the facts about the identity, physical properties, and physiological actions of PP, this review will help to at least reinstate it as a potential anesthetic agent for experimental research and other types of non-applied investigations. Due to the possible effects of elevated Mg^2+^ levels on echinoderm MCT, it is also preferable, in our present state of knowledge, to MgCl_2_—the most frequently cited echinoderm anesthetic—for experimental work involving isolated preparations that include collagenous components such as ligaments, tendons, and dermal tissue.

Regarding the commercial exploitation of edible sea cucumbers and sea urchins, MgCl_2_-induced anesthesia is currently a favored method for increasing their resistance to stress caused by handling and transportation and for facilitating aquaculture and fishery research [153,154,155,156,157]. It has been reported, however, that MgCl_2_-induced anesthesia does not prevent sea cucumber dermal autolysis [158]. Autolysis—the enzymolytic disaggregation of the collagenous dermis of the body wall—occurs in response to a range of external stimuli, such as exposure to sunlight, temperature changes, and mechanical damage. It reduces the quality and nutritional value of sea cucumbers and causes significant economic losses for the industry. Although autolysis can be pre-empted by freezing, boiling or drying, fresh animals kept in seawater are more attractive economically and gastronomically, which has stimulated a search for suitable autolysis inhibitors [159,160]. Hill’s evidence that PP blocks autolysis [85,88] (Section 3.2.1) was derived mainly from a single experiment involving subjective quantification and requires verification. However, this evidence does suggest that it may be worth exploring the potential of PP as an anesthetic for employment in sea cucumber and sea urchin research methodologies and production chains. There would seem, for example, to be scope for its use in post-harvest stages such as grading of stock and transportation to and between culture and holding facilities, though not, at present, to markets: PP has been found to exhibit low acute toxicity but has not been tested for carcinogenicity and, with regard to human health, has been recommended as “a candidate for further work” [161]. PP is as readily procurable as MgCl_2_, and the cost per unit volume of anesthetic formulation is, according to the present author’s estimate, around the same for both agents (assuming aquarium-grade MgCl_2_) and much cheaper than that for MS-222, which has also been promoted as an echinoderm anesthetic [113,116,162]. Table 2 summarizes available information on methodological aspects of PP, MgCl_2,_ and MS-222 as anesthetics for echinoderms and indicates deficiencies in current knowledge.

### 4.3. Suggestions for Further Work

With regard to all potential applied and non-applied uses of PP, there is a need to determine optimal concentrations required to achieve acceptable induction and recovery times and to establish criteria for different endpoints, including immobilization (sedation), muscle relaxation, deep anesthesia, and full recovery from these states, the ultimate aim being to establish standardized protocols. The importance of evaluating concentration-dependent responses, as well as longer-term post-exposure outcomes, is illustrated by Mercer et al. [164]. Ideally, the subjects of such methodological studies would be widely utilized research models and commercially important species, such as (representing each echinoderm class) *Asterias forbesi*, *Antedon mediterranea*, *Paracentrotus lividus*, *Apostichopus japonicus* and *Amphiura filiformis*. The evaluation of PP should also be extended to include physiological stress responses [113].

To permit an informed assessment of the relative merits of PP and MgCl_2_ as suitable anesthetics for experimental work on echinoderms, the possible contribution of Mg^2+^ ions to the structural integrity of MCT requires exploration. As the evidence for such a role was derived solely from sea cucumber dermis [124,125], which may not be a representative MCT [115], investigations should include a range of mutable collagenous structures, ideally from all five classes, with the aim of characterizing and quantifying any effect and evaluating the extent to which this should influence the use of MgCl_2_.

Before PP is utilized as an anesthetic for edible sea cucumbers and sea urchins, it would be advisable to determine if it affects their gastronomic quality. It is surprising that such investigations appear not to have been conducted on anesthetic agents currently used on these animals, particularly MgCl_2_, which has been found to change the taste and texture of foods [165,166], and MS-222, which can indirectly improve the eating quality of fish by reducing stress during transportation [167,168].

In common with most chemical agents applied to aquatic animals [169], the toxicological properties of PP are still to be fully characterized [161]. Whilst not restricting its use in non-applied research, this limits its range of potential applications in aquaculture and fisheries to procedures chronologically remote from the preparation of processed products or the sale of fresh unprocessed products. First steps might be to determine the dynamics of PP contamination of sea cucumber body wall and sea urchin gonads after anesthesia and relate this information to acceptable daily intake data obtained by standard laboratory animal testing. It is hoped that the growing ethical concerns about the treatment of echinoderms and other invertebrates in the laboratory and in the food production industry [170] will eventually generate the incentive and funding needed to accomplish these and the preceding suggested research objectives.

## Figures and Tables

**Table 1:** Examples of the use of PP as an echinoderm anesthetic, both for whole animals and for isolated tissue preparations. In all examples, PP was used as a 0.1% solution in natural or artificial seawater, and the sources of PP were Nipa Laboratories or Sigma-Aldrich, except regarding ref. [90], which did not cite the source but whose “PP” was confirmed by mass spectrometry to have a molecular weight of 152.19 [116], which is compatible with 1-phenoxypropan-2-ol.

Class and Species	PP Used to Anesthetize:		Reference
	Whole Animal	Isolated Tissue Preparation	
Asteroidea			
*Asterias rubens*	Yes	Aboral body wall	[55]
*Marthasterias glacialis*	Yes	No	[62]
Crinoidea			
*Cenocrinus asterius*	No	Stalk ligaments	[65]
*Antedon mediterranea*	Yes	No	[70]
Echinoidea			
*Echinus esculentus*	No	Compass rotular ligaments	[80]
*Paracentrotus lividus*	No	Compass depressor ligaments	[83]
Holothuroidea			
*Eupentacta quinquesemita*	Yes	Introvert body wall	[90]
*Holothuria forskali*	Yes	No	[101]
Ophiuroidea			
*Amphipholis squamata*	Yes	No	[105]
*Ophiura ophiura*	Yes	Oral arm plate ligaments	[109]

**Table 2:** Methodological aspects of PP, MgCl_2_ and MS-222 as anesthetic agents for echinoderms. The cost comparison assumes aquarium-grade MgCl_2_. GBP, British pound (£). ^1^ Possible inhibition of sea cucumber dermal autolysis. ^2^ Possible influence on structural integrity of MCT. Assumed formulations are: ^3^ 0.1% in seawater; ^4^ 1:1 seawater and 370 mmol L^−1^ MgCl_2_; ^5^ 0.8 g L^−1^ in seawater.

Aspect	PP	MgCl_2_	MS-222	Reference
Standardized protocols	No	No	No	-
Information on efficacy	No	Yes	Yes	[113]
Uncertain non-anesthetic actions	Yes ^1^	Yes ^2^	No	[124,125]
Human toxicity concerns	Yes	No	Yes	[160,163]
Solubility and dissolution rate	+	++	++	-
Commercial availability	++	++	++	-
Cost L^−1^ of formulation (GBP)	ca. 0.03 ^3^	ca. 0.03 ^4^	ca. 3.00 ^5^	-

## Data Availability

Data are contained within the article or Supplementary Material.

## Supplementary Materials

The following supporting information can be downloaded at https://www.mdpi.com/article/10.3390/biology15212007/s1. Table S1: Publications (1900-2026) reporting investigations that refer to the use of propylene phenoxetol to successfully induce anesthesia in echinoderm subjects (completed 14th September 2026).
