# Supplementary material for: Propylene Phenoxetol (1-Phenoxypropan-2-ol): Its Use as an Anesthetic for Echinoderms
Source: Biology (Basel). 2026 Nov 5;15(21):2007. doi: 10.3390/biology15212007 (PMC13761573; doi:10.3390/biology15212007)
Supplement: Supplementary file 1 [file biology-15-02007-s001.zip › biology-4633775-supplementary.pdf]

Table S1. Publications (1900-2026) reporting investigations that refer to the use of propylene phenoxetol to successfully induce anesthesia in echinoderm subjects (completed 14th September 2026). The references for each class are arranged in chronological order with the earliest at the top and latest at the bottom. Full reference citations are provided in the 'Reference' section of the review. Asterisks indicate binomials updated via WoRMS (World Register of Marine Species; https://www.marinespecies.org/) and therefore differing from the versions provided in the cited references. IP, isolated preparation; IT, immersion time in anesthetic solution; MCT, mutable collagenous tissue; PP, propylene phenoxetol (verified); "PP", propylene phenoxetol (unverified); RT, immersion time in anesthetic-free medium to permit recovery; Satd., saturated; WA, whole animal.

Class and species	Investigation category	PP	"PP"	Concn. %	IT (min)	RT (min)	IP/WA	Reference
**Asteroidea**								
Echinaster echinophorus	Morphology	■		0.15			WA	[54]
Asterias rubens	MCT physiology	■		0.1	30-60		IP+WA	[55]
Asterias rubens	Regeneration	■		0.1	30	15-60	WA	[56]
Asterias rubens, Marthasterias glacialis	Morphology	■		0.1			WA	[57]
Pycnopodia helianthoides	MCT physiology	■		0.1	30-60	≥50	IP+WA	[58]
Asterias rubens	Regeneration		■	0.1			WA	[59]
Asterias rubens	Other physiology	■		0.1	60		WA	[60]
Marthasterias glacialis	MCT physiology	■		0.1	60		WA	[61]
Asterias rubens, Marthasterias glacialis	Morphology	■		0.1	60		WA	[62]
**Crinoidea**								
Antedon bifida	Morphology		■				WA	[52]
Antedon bifida	MCT physiology	■		0.1	15-20	30-45	IP	[63]
Cenocrinus asterius	MCT physiology	■		0.1			IP	[64]
Cenocrinus asterius	MCT physiology	■		0.1	30		IP	[65]
Antedon mediterranea	MCT physiology	■		0.1			IP	[66]
Metacrinus rotundus	MCT physiology	■		0.1			IP	[67]
Antedon mediterranea	Other physiology	■		0.1			WA	[68]
Antedon mediterranea	Other physiology	■		0.1		60	WA	[69]
Antedon mediterranea	Other physiology	■		0.1			WA	[70]
**Echinoidea**								
Echinus esculentus	Morphology	■					IP	[53]
Echinocardium cordatum	Morphology	■		0.1			IP	[71]
Sphaerochinus granularis	Morphology	■		0.1			WA	[72]
Paracentrotus lividus	MCT physiology	■		0.1	30		IP	[73]
Echinus esculentus	MCT physiology	■		0.1			IP	[74]
Paracentrotus lividus	MCT physiology	■		0.1	30		IP	[75]
Paracentrotus lividus	Morphology	■		0.1			WA	[76]
Stylocidaris affinis	MCT physiology	■		0.1	30-45	45	IP	[77]
Stylocidaris affinis	Other physiology	■		0.1	30	20	IP	[78]
Stylocidaris affinis	Other physiology	■		0.1	40	30	IP	[79]
Echinus esculentus	Other physiology	■		0.1			IP	[80]
Paracentrotus lividus	MCT physiology	■		0.1	45		IP	[81]
Paracentrotus lividus	MCT physiology	■		0.1	45		IP	[82]
Paracentrotus lividus	MCT physiology	■		0.1	45		IP	[83]
Paracentrotus lividus	MCT physiology	■		0.1	45		WA	[84]
**Holothuroidea**								
*Isostichopus badionotus**	Other physiology		■				WA	[85]
*Pseudocolochirus violaceus**	Other physiology	■		0.25	15	40-60	WA	[86]
*Sclerodactyla briareus**	Other physiology		■				WA	[87]
Isostichopus badionotus	Other physiology		■	Satd.			WA	[88]
Isostichopus badionotus	Morphology		■				WA	[89]
Eupentacta quinquesemita	MCT physiology	■		0.1	180-300		IP+WA	[90]
Eupentacta quinquesemita	Other physiology	■		0.1	180-300		WA	[91]
Holothuria forskali	Other physiology	■		0.2	60		WA	[92]
Holothuria scabra	Symbiont biology	■		2			WA	[93]
Holothuria forskali	Morphology	■		0.1			WA	[94]
Holothuria forskali	Morphology	■		0.2			WA	[95]
Holothuria tubulosa	Morphology	■		0.2	15		WA	[96]
Leptosynapta galliennii	Morphology	■			60		WA	[97]
Holothuria forskali	Regeneration	■		0.2	60		WA	[98]
Isostichopus badionotus	MCT physiology	■		Satd.			WA	[99]
Synapta maculata	Morphology	■		0.1			WA	[100]
Holothuria forskali	MCT physiology	■		0.1	60		WA	[101]
Holothuria forskali	MCT physiology	■		0.1			WA	[102]
**Ophiuroidea**								
Acrocnida brachiata	Other physiology		■	1			WA	[103]
Ophiocomina nigra	MCT physiology	■		0.1	20-30		IP	[104]
Amphipholis squamata	Behaviour	■		0.1	30-40		WA	[105]
Ophiocomella ophiactoides	Reproductive biology	■		1			WA	[106]
Ophiocomella ophiactoides	Reproductive biology	■		0.1	10-20		IP+WA	[107]
Ophiocomella ophiactoides	Symbiont biology	■		0.1			WA	[108]
Ophiura ophiura	MCT physiology	■		0.1	30		IP+WA	[109]
Ophioderma longicaudum	Other physiology	■		0.1			IP	[110]
Ophiozonella stellamaris	Taxonomy		■	0.15			WA	[111]
Ophiocomina nigra	Morphology	■		0.1	30		WA	[112]
